# Supplementary material for: Leukemia circulation kinetics revealed through blood exchange method
Source: Commun Biol. 2024 Apr 20;7:483. doi: 10.1038/s42003-024-06181-x (PMC11032325; doi:10.1038/s42003-024-06181-x)
Supplement: Supplementary file 2 — Description of Additional Supplementary Files [file 42003_2024_6181_MOESM2_ESM.pdf]

Description of Additional Supplementary Files

File name: Supplemental Data
Description: The source data behind the graphs in the paper.
