# Supplementary material for: Leukemia circulation kinetics revealed through blood exchange method
Source: Commun Biol. 2024 Apr 20;7:483. doi: 10.1038/s42003-024-06181-x (PMC11032325; doi:10.1038/s42003-024-06181-x)
Supplement: Supplementary file 4 — Reporting Summary [file 42003_2024_6181_MOESM4_ESM.pdf]

nature portfolio

Corresponding author(s): Scott Manalis

updated by author(s): March 24, 2024

Reporting Summary

Nature Portfolio wishes to improve the reproducibility of the work that we publish. This form provides structure for consistency and transparency in reporting. For further information on Nature Portfolio policies, see our Editorial Policies and the Editorial Policy Checklist.

Statistics

For all statistical analyses, confirm that the following items are present in the figure legend, table legend, main text, or Methods section.

n/a	Confirmed	
☐	☒	The exact sample size (*n*) for each experimental group/condition, given as a discrete number and unit of measurement
☐	☒	A statement on whether measurements were taken from distinct samples or whether the same sample was measured repeatedly
☐	☒	The statistical test(s) used AND whether they are one- or two-sided<br>*Only common tests should be described solely by name; describe more complex techniques in the Methods section.*
☒	☐	A description of all covariates tested
☐	☒	A description of any assumptions or corrections, such as tests of normality and adjustment for multiple comparisons
☐	☒	A full description of the statistical parameters including central tendency (e.g. means) or other basic estimates (e.g. regression coefficient) AND variation (e.g. standard deviation) or associated estimates of uncertainty (e.g. confidence intervals)
☐	☒	For null hypothesis testing, the test statistic (e.g. F, t, r) with confidence intervals, effect sizes, degrees of freedom and P value noted<br>*Give P values as exact values whenever suitable.*
☒	☐	For Bayesian analysis, information on the choice of priors and Markov chain Monte Carlo settings
☐	☒	For hierarchical and complex designs, identification of the appropriate level for tests and full reporting of outcomes
☐	☒	Estimates of effect sizes (e.g. Cohen's *d*, Pearson's *r*), indicating how they were calculated

Our web collection on statistics for biologists contains articles on many of the points above.

Software and code

Policy information about availability of computer code

Data collection	LabVIEW 2017 Version 17.0, NIS-Elements Advanced Research version 4.20
Data analysis	MATLAB R2017b Version 9.3.0.713579, Microsoft Excel 2016,

For manuscripts utilizing custom algorithms or software that are central to the research but not yet described in published literature, software must be made available to editors and reviewers. We strongly encourage code deposition in a community repository (e.g. GitHub). See the Nature Portfolio guidelines for submitting code & software for further information.

Data

Policy information about availability of data

All manuscripts must include a data availability statement. This statement should provide the following information, where applicable:

- Accession codes, unique identifiers, or web links for publicly available datasets
- A description of any restrictions on data availability
- For clinical datasets or third party data, please ensure that the statement adheres to our policy

Data Availability Statement:
The data that support the findings of this study are available from the corresponding authors upon reasonable request.

Field-specific reporting

Please select the one below that is the best fit for your research. If you are not sure, read the appropriate sections before making your selection.

☒ Life sciences ☐ Behavioural & social sciences ☐ Ecological, evolutionary & environmental sciences

For a reference copy of the document with all sections, see nature.com/documents/nr-reporting-summary-flat.pdf

Life sciences study design

All studies must disclose on these points even when the disclosure is negative.

Sample size

No sample size calculation was performed. Based on previous work, our goal was to have at least four independent replicates for each experiment in order to perform statistical analysis.

Data exclusions

No data was excluded

Replication

All attempts at replication were successful.

Each blood exchange experiment was performed with an independent pair of donor/recipient mice on separate days.

For the ALL model, the number of mice were as follows:
- RFP+ tumor bearing donor with healthy recipient: 7 pairs of mice
- RFP+ tumor bearing donor with RFP- tumor bearing recipient: 6 pairs of mice
- RFP+ tumor bearing donor with treated RFP- tumor bearing recipient: 6 pairs of mice
- RFP+ tumor bearing donor with E-selectin antibody treated healthy recipient: 4 pairs of mice
- Relapse RFP+ tumor bearing donor with healthy recipient: 4 pairs of mice
- RFP+ tumor bearing donor with chemotherapy treated healthy recipient: 4 pairs
- RFP+ tumor bearing donor with irradiated healthy recipient: 4 pairs

For the AML model, the number of mice were as follows:
- RFP+ tumor bearing donor with healthy recipient: 9 pairs of mice
- Relapse RFP+ tumor bearing donor with healthy recipient: 5 pairs of mice
- Relapse RFP+ tumor bearing recombinant E-selectin treated donors with healthy recipient: 5 pairs of mice

Randomization

Blinding

Blinding was not performed for setups in blood exchange. Blinding was not needed for analysis because all samples were analyzed in the same way and objective readouts were used

Reporting for specific materials, systems and methods

We require information from authors about some types of materials, experimental systems and methods used in many studies. Here, indicate whether each material, system or method listed is relevant to your study. If you are not sure if a list item applies to your research, read the appropriate section before selecting a response.

Materials & experimental systems

n/a	Involved in the study
☐	☒ Antibodies
☐	☒ Eukaryotic cell lines
☒	☐ Palaeontology and archaeology
☐	☒ Animals and other organisms
☒	☐ Human research participants
☒	☐ Clinical data
☒	☐ Dual use research of concern

Methods

n/a	Involved in the study
☒	☐ ChIP-seq
☐	☒ Flow cytometry
☒	☐ MRI-based neuroimaging

Antibodies

Antibodies used

Staining antibodies (mouse)
CD31 (endothelial marker) [BioLegend: 102410]

CD45 (white blood cell marker) [BioLegend: 103108]
E-selectin [Santa Cruz Biotechnology: sc-59766 PE]
VCAM1 [Life Technologies: 11-1061-82]
Integrin β1 [Life Technologies: 11-0291-82]
CD45 (white blood cell) [BioLegend: 103114]

For E-selectin binding assay:
recombinant E-selectin human IgG chimera [BioLegend: 755504]
α-Human IgG [Life Technologies: A10631]

Isotype controls:
Rat IgG Isotype PE [Life Technologies: 12-4031-81]
Rat IgG Isotype FITC [Life Technologies: 11-4321-85]

Validation	Antibodies were validated by manufacturer for the species and application of this study and supported by publication.<br>See manufacturer website for validation statements (www.biolegend.com, www.scbt.com, www.thermofisher.com)<br>All experiments included appropriate use of isotype control

Eukaryotic cell lines

Policy information about cell lines

Cell line source(s)	Cell line established in the lab of Michael Hemann
Authentication	no authentication method used
Mycoplasma contamination	All cell lines tested negative for mycoplasma contamination
Commonly misidentified lines<br>(See ICLAC register)	No commonly misidentified cell lines were used in this study

Animals and other organisms

Policy information about studies involving animals; ARRIVE guidelines recommended for reporting animal research

Laboratory animals	Mice were 12-16 weeks at the time of tumor initiation. C57BL/6J mice were used. Only male mice were used, as cell lines were both derived from male mice and do not effectively engraft in female mice
Wild animals	Study did not involve wild animals
Field-collected samples	Study did not involve field-collected samples
Ethics oversight	All animal-based procedures were approved by the Massachusetts Institute of Technology Committee on Animal Care (CAC), Division of Comparative Medicine (DCM).

Note that full information on the approval of the study protocol must also be provided in the manuscript.

Flow Cytometry

Plots

Confirm that:

☒ The axis labels state the marker and fluorochrome used (e.g. CD4-FITC).

☒ The axis scales are clearly visible. Include numbers along axes only for bottom left plot of group (a 'group' is an analysis of identical markers).

☒ All plots are contour plots with outliers or pseudocolor plots.

☒ A numerical value for number of cells or percentage (with statistics) is provided.

Methodology

Sample preparation	Bone marrow cells were isolated through manual grinding of left and right femurs with mortar and pestle, and red blood cells removed through ACK lysis buffer before 10 minutes FcX blocking and 20 minutes of staining at 4°C. Blood samples were collected from terminal cardiac puncture, and red blood cells were removed with two rounds of ACK lysis buffer before 10 minutes FcX blocking and 20 minutes of staining at 4°C.
Instrument	Flow cytometry was performed on a BD LSR HTS-2 analyzer
Software	BD FACSDIVA was used to collect the data. Data analysis was performed using FlowJo X 10.0.7r2
Cell population abundance	Bone marrow contained 30-95% leukemia tumor cells (constituitively RFP+) and <1% endothelial cells, blood contained

Cell population abundance	10-50% leukemia tumor cells.
Gating strategy	Bone marrow endothelial cell adhesion molecules:<br>After selecting for single cells through forward and side scatter, endothelial cells were selected as DAPI-, CD31+, and CD45-. A cutoff on the endothelial cells in the channel of the adhesion molecule antibody (E-selectin PE or VCAM1 FITC) was used to identify high expressing cells within the BMECs. Because of the low abundance of endothelial cells in the marrow <1%, at least 1 million cells were analyzed per mouse bone marrow sample.<br><br>Adhesion marker expression on leukemia cells:<br>After selecting for single cells through forward and side scatter, tumor cells were selected as DAPI-, CD45+, PE+ (constitutively expressed RFP). Average expression in the channel of the adhesion molecule (E-selectin binding-FITC or integrin β1-FITC) was then used to assess binding potential.

☒ Tick this box to confirm that a figure exemplifying the gating strategy is provided in the Supplementary Information.
